# Graves' Disease Presenting with Complete Atrioventricular Block

**DOI:** 10.1155/2020/6656875

**Published:** 2020-12-15

**Authors:** Young Sil Eom, Pyung Chun Oh

**Affiliations:** Department of Internal Medicine, Gil Medical Center, Gachon University College of Medicine, Incheon, Republic of Korea

## Abstract

Hyperthyroidism commonly causes tachyarrhythmias such as sinus tachycardia and atrial fibrillation. Impaired atrioventricular conduction is a very rare complication of hyperthyroidism. We report a case of a patient with hyperthyroidism due to Graves' disease presenting with syncope and complete atrioventricular block. Because lack of awareness of atypical presentation in patients with hyperthyroidism may delay diagnosis and treatment, the recognition that hyperthyroidism can be one of the reversible causes of complete atrioventricular block is important.

## 1. Introduction

Although thyrotoxicosis commonly causes tachyarrhythmias including sinus tachycardia and atrial fibrillation, atrioventricular (AV) block is very rarely described in patients with hyperthyroidism. Here, we report a case of a patient who presented with syncope due to complete AV block, was diagnosed with Graves' disease, and completely recovered on antithyroid treatment.

## 2. Case Presentation

**Figure 1:**
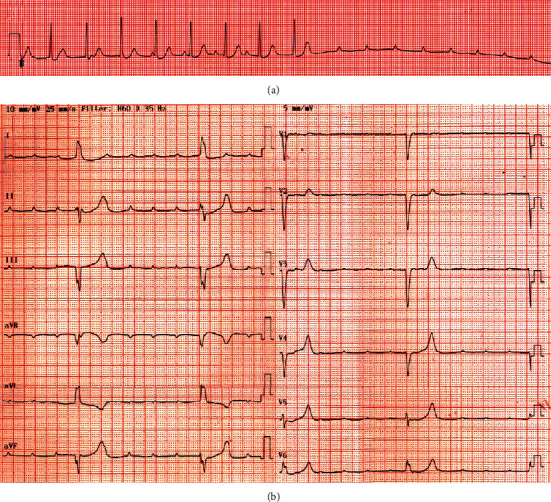
(a) Electrocardiography performed at the primary care clinic showing complete atrioventricular block with supraventricular rhythm, followed by long ventricular asystole over 4.4 seconds. (b) Follow-up electrocardiography at the emergency room revealing complete AV block with ventricular escape rhythm.

**Figure 2:**
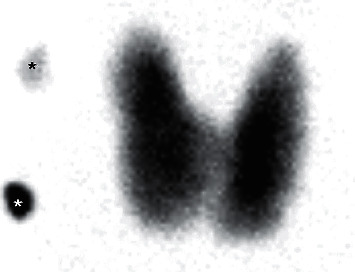
Radioiodine scan showing intense increased thyroid uptake, consistent with hyperthyroidism.

**Figure 3:**
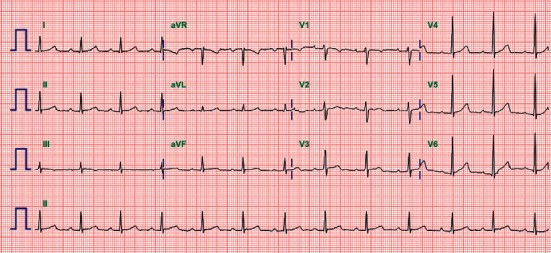
8-day follow-up electrocardiography after decreasing the level of free T4 and T3 with propylthiouracil showing sinus rhythm.

A 28-year-old female patient had presented with dizziness a week ago. While she was waiting for medical consultation at a primary care clinic, she suddenly lost consciousness and had involuntary convulsive-like movements for 10 seconds. She had a history of acute otitis media 7 days ago and had taken medications including antibiotics for 3 days. The electrocardiogram performed at the primary care clinic showed complete AV block with supraventricular rhythm, followed by long ventricular asystole over 4.4 seconds (Figure 1(a)). Initial blood pressure and pulse rate were 128/70 mmHg and 30 per minute, respectively. Follow-up electrocardiography at the emergency room revealed complete AV block with the ventricular escape rhythm (Figure 1(b)). A temporary transvenous ventricular pacing was applied through the internal jugular vein. Serum electrolyte levels were within the normal clinical range, and renal function was normal. She did not take other or herbal medications except for drugs for acute otitis media including cefditoren, erdosteine, and levocetirizine. The thyroid gland was diffusely enlarged, and bilateral exophthalmos was identified. The level of the thyroid-stimulating hormone was very low, less than 0.008 *μ*IU/mL (reference range, 0.55–4.78 *μ*IU/mL), and the levels of free T4 and T3 were 6.4 ng/dL and 511.0 ng/dL, respectively (reference range, 0.89–1.78 ng/dL and 60–180 ng/dL, respectively). Propylthiouracil 300 mg daily was started. Thyroid ultrasound noted diffuse thyroid enlargement with heterogenous parenchymal echogenicity. Radioiodine scan showed intense increased thyroid uptake, consistent with hyperthyroidism (Figure 2). Echocardiography reported normal cardiac function without structural abnormalities. Coronary computed tomography angiography or cardiac catheterization was not performed because she did not have any risk factors for coronary artery disease, and myocardial ischemia was less likely to be the cause of complete AV block in this patient. At the eighth hospital day, the levels of free T4 and T3 decreased to 2.9 ng/dL and 222.3 ng/dL, respectively. Complete AV block was recovered to sinus rhythm with rates of 75 per minute (Figure 3), and transvenous pacing lead was removed. She was discharged with medications for hyperthyroidism without the recurrence of the AV block.

## 3. Discussion

Complete AV block results from various pathologic states causing an anatomical or functional impairment in the conduction system. The common causes of complete AV block include degenerative diseases characterized with progressive fibrosis of the conduction system, cardiac diseases such as ischemic heart disease or cardiomyopathies, and medications impairing AV conduction. In addition, electrolyte disturbance (especially, hyperkalemia) or thyroid disease can lead to AV block.

Thyroid hormones are known to have direct inotropic, chronotropic, and dromotropic effects such as tachycardia and increased cardiac output. Therefore, hyperthyroidism commonly causes tachyarrhythmias such as sinus tachycardia and atrial fibrillation, and bradyarrhythmias occur more commonly in association with hypothyroidism. Complete atrioventricular block can be present in patients with hypothyroidism, which resolves after levothyroxine therapy [1]. In addition, thyrotoxic periodic paralysis rarely causes AV block, which is related with hypokalemia [2].

Impaired AV conduction is a very rare complication of thyrotoxicosis [3, 4]. Bradyarrhythmias complicating hyperthyroidism have been commonly associated with acute infectious disease, hypercalcemia, thyroid storm, or underlying structural heart disease [5–7]. In our patient, acute otitis media had been preceded before the development of complete AV block. The exact mechanism of complete AV block in thyrotoxicosis has not been elucidated. Interstitial inflammation of the AV node or focal myocarditis around the AV node has been reported in a patient with hyperthyroidism [8, 9]. Autopsies in patients with fatal hyperthyroidism showed myocyte hypertrophy, myocyte necrosis, interstitial and perivascular fibrosis, and myocardial edema, which has been postulated to result in AV block [10].

In conclusion, we report a case of a patient with hyperthyroidism due to Graves' disease presenting with syncope and complete AV block. Complete AV block rarely occurs in thyrotoxicosis. Because lack of awareness of atypical presentations in patients with hyperthyroidism may delay diagnosis and treatment, the recognition that hyperthyroidism can be a reversible cause of complete AV block is important.

## Data Availability

The data used and/or analyzed during the current study are available from the corresponding author upon request.
